# Protection of α-CaMKII from Dephosphorylation by GluN2B Subunit of NMDA Receptor Is Abolished by Mutation of Glu^96^ or His^282^ of α-CaMKII

**DOI:** 10.1371/journal.pone.0162011

**Published:** 2016-09-09

**Authors:** Madhavan Mayadevi, Kesavan Lakshmi, Sudarsana Devi Suma Priya, Sebastian John, Ramakrishnapillai V. Omkumar

**Affiliations:** Molecular Neurobiology Division, Rajiv Gandhi Centre for Biotechnology, Thycaud, P. O., Thiruvananthapuram-695014, Kerala, India; University of Alabama at Birmingham, UNITED STATES

## Abstract

Interaction of CaMKII and the GluN2B subunit of NMDA receptor is essential for synaptic plasticity events such as LTP. Synaptic targeting of CaMKII and regulation of its biochemical functions result from this interaction. GluN2B binding to the T-site of CaMKII leads to changes in substrate binding and catalytic parameters and inhibition of its own dephosphorylation. We find that CaMKIINα, a natural inhibitor that binds to the T-site of CaMKII, also causes inhibition of dephosphorylation of CaMKII similar to GluN2B. Two residues on α-CaMKII, Glu^96^ and His^282^, are involved in the inhibition of CaMKII dephosphorylation exerted by binding of GluN2B. E96A-α-CaMKII is known to be defective in GluN2B-induced catalytic modulation. Data presented here show that, in both E96A and H282A mutants of α-CaMKII, GluN2B-induced inhibition of dephosphorylation is impaired.

**Highlights**

- CaMKIINα inhibits dephosphorylation of CaMKII
- Glu^96^ and His^282^ of α-CaMKII mediate the GluN2B induced regulatory effects

## 1. Introduction

Many mechanisms exist for the stable storage of information in living systems. Activity dependent strengthening of neuronal synapse leads to long term potentiation (LTP), a cellular mechanism required for learning and memory. The activation of NMDA receptor (NMDAR) and subsequent influx of calcium into the postsynaptic compartment activates calcium/calmodulin dependent protein kinase II (CaMKII) and initiates downstream events required for the induction of LTP. CaMKII is a dodecameric holoenzyme that can undergo autophosphorylation generating calcium independent activity [1]. The autophosphorylation of CaMKII at Thr^286^ is important for the induction of LTP [2–6]. It has been suggested that CaMKII in concert with protein phosphatase 1 (PP1) can act as a bistable switch [7,8]. Reconstitution of CaMKII-Thr^286^ autophosphorylation *in vitro* in presence of PP1 and GluN2B sequence shows that the presence of GluN2B favors the phosphorylated state of Thr^286^ [9] and generates a system that shows biochemical properties necessary for a memory switch [8]. The autophosphorylated CaMKII bound to postsynaptic density (PSD) can be dephosphorylated by PP1 in PSD [10,11]. The CaMKII-phosphatase system can function as a molecular switch which is energy efficient and is sensitive to calcium signals and can aid in the formation of stable memories [9–15]. CaMKII as it translocates to PSD binds to GluN2B subunit of NMDAR [16–19]. The interaction of CaMKII with GluN2B is important for induction and maintenance of LTP [20–22]. The disruption of this interaction causes impairment of LTP [23,24]. Further, the binding of GluN2B to the T-site of CaMKII converts the enzyme into a persistently active state, and also modulates the catalytic activity of the enzyme, by altering the parameters of kinetics and substrate binding [9,25–27]. The interaction of CaMKII with GluN2B thus contributes to the switch that supports memory maintenance [8,9,28–30].

CaMKII inhibitor protein CaMKIINα is an endogenous inhibitor of CaMKII, which binds to the T-site of CaMKII [31–34]. The CaMKIINα mRNA is upregulated during fear learning [35, 36]. Sufficient concentration of a CaMKIINα derived peptide, CaMKIINtide, disrupted the CaMKII-NMDAR complex and caused a persistent reduction in the complex, leading to reduction in synaptic strength as seen by the depotentiation and the reversal of LTP maintenance [21,22].

The current study probes the impact of T-site binding proteins on the dephosphorylation of CaMKII. An attempt has been made to decipher amino acid residues of CaMKII involved in the structural changes accompanying the binding of ligands to the T-site of CaMKII.

## 2. Materials and Methods

### 2.1. Materials

ATP, calmodulin, calmodulin-agarose, protease inhibitor cocktail, anti-α-CaMKII antibody, secondary antibody conjugates, PMSF (phenylmethylsulfonylfluoride) and DTT (dithiothreitol) were from Sigma Chemicals, USA. Phosphocellulose was from Whatman, UK. IPTG (Isopropylthiogalactoside) and glutathione sepharose 4B were from GE, USA. Pierce glutathione-agarose was from Thermo Fisher Scientific. Phospho-Thr^286^-α-CaMKII antibody, was either from Sigma-Aldrich or from Cell Signaling Technology. Oligonucleotides were obtained from SigmaGenosys, USA. Quikchange site directed mutagenesis kit was from Stratagene, USA. Nitrocellulose paper was from PALL Gelmann. [γ-^32^P]ATP was from Bhabha Atomic Research Centre, India. Anti-glutathione-S-transferase (GST) antibody was from Santacruz Biotechnology Inc., USA. Protein phosphatase 1 (PP1) was from New England Biolabs, USA. GST-CaMKIINα plasmid was a gift from Dr. P. Rangarajan, Department of Biochemistry, Indian Institute of Science, Bangalore, India.

### 2.2. Preparation of CaMKII

WT-α-CaMKII and E96A-α-CaMKII mutant were expressed in Sf21 or High Five insect cells. The crude insect cell lysate and the purified enzymes were prepared as described earlier [27,37,38].

GFP-α-CaMKII expressed in HEK-293 cells was also used in the experiments. WT and mutants, H282A and K21A, of GFP-α-CaMKII were used. The cells were lysed in RIPA buffer or in solution containing 50 mM Pipes (pH 7.0), 2 mM DTT, 1 mM PMSF and protease inhibitor cocktail.

### 2.3. Expression of fusion proteins

CaMKIINα inhibitor protein, S1291A-GluN2A mutant (with amino acid sequences 1265-1301of GluN2A) and S1303A-GluN2B mutant (with amino acid sequence 1271-1311of GluN2B) were expressed in BL21(DE3)pLys strain of *E*. *coli* cells as GST fusion proteins. S1291A-GluN2A and S1303A-GluN2B mutants are the non-phosphorylatable forms of GluN2A and GluN2B sequences. These non-phosphorylatable forms are used to rule out any substrate phosphorylation occurring during the reaction and interfering with the binding. The expression and purification were carried out as published earlier [27,38]. The expressed proteins were purified using glutathione-sepharose column and were subsequently subjected to gel filtration to remove glutathione. S1 Fig shows the preparations of GST-S1291A-GluN2A, GST-S1303A-GluN2B and GST-CaMKIINα. GluN2B sequence containing the amino acids 1271–1311 was cloned into pET-32a vector to obtain His-GluN2B with His tag expressed at the C-terminal.

The concentrations of proteins were estimated using BCA (Bicinchoninic acid) method [39].

### 2.4. GST pulldown assay

CaMKII was incubated with either GST-GluN2B or GST-CaMKIINα or GST-GluN2A in binding buffer (50 mM Pipes, pH 7.0, 0.1% BSA, 150 mM NaCl and 0.1% Tween-20) either in presence or absence of 1 mM CaCl_2_ and 1 μM calmodulin for 1 hour at 4°C. The reaction mix was allowed to bind for 1 hour at 4°C to glutathione-sepharose beads which were washed in PBS (10 mM disodium hydrogen phosphate, 1.8 mM potassium dihydrogen orthophosphate, 0.14 M sodium chloride, 2.7 mM potassium chloride, pH 7.4). Alternatively, PBS washed glutathione-sepharose beads were first incubated with the GST-fusion proteins in PBS for 1 hour at 4°C, washed 4 times with PBS and were subsequently incubated with CaMKII in binding buffer for 1 hour at 4°C. After incubation, the glutathione-sepharose beads were again washed with PBS four times and were suspended in SDS sample buffer. The samples were heated in a boiling water bath for 4 minutes and were subjected to SDS-PAGE, followed by Western blot analysis using anti-α-CaMKII antibody and anti-GST antibody [40,41]. Blots were developed either by colour reaction of alkaline phosphatase conjugated secondary antibody or by enhanced chemiluminescence using horse radish peroxidase conjugated secondary antibody. Densitometric quantitation of the blots were done after capturing images of the blots, using BioRad Quantity One software. In competition experiments, CaMKII was incubated with His-GluN2B and GST-CaMKIINα simultaneously.

In the pulldown assay of WT and E96A of α-CaMKII purified after expression in insect cells and WT, H282A and K21A expressed as GFP-α-CaMKII in HEK-293 cells, GST-GluN2B was first bound with washed glutathione-agarose beads. The GST-GluN2B bound beads were washed 4 times with PBS. Equal amounts of GST-GluN2B bound beads were allowed to bind to the WT or mutants of CaMKII. The autophosphorylated CaMKII samples for pulldown were prepared as described in the section 2.5 below except for using nonradioactive ATP. The pulldown was done under the same reaction conditions as that of the dephosphorylation experiments.

### 2.5. Autophosphorylation of Thr^286^-α-CaMKII and its dephosphorylation

Autophosphorylation of CaMKII was carried out in a reaction mix containing 50 mM Tris, pH 8.0, 10 mM MgCl_2_, 10 mM DTT, 1 μM γ^32^P-ATP, 3 mM CaCl_2_, 9 μM calmodulin and 0.164 mg/ml of purified WT-α-CaMKII. The reaction mix was preincubated at 30°C for 1 min and autophosphorylation was carried out for 30 seconds after initiating the reaction with ATP addition. The reaction was stopped with either SDS-PAGE sample buffer or with 10 μM staurosporine. For dephosphorylation experiments, the reaction mix after staurosporine addition was incubated with either 10 μM GST or 5.7 μM GST-CaMKIINα in presence of 22 μM calmodulin and 1 mM CaCl_2_ for 20 minutes at 4°C. GST was used as control for GST-CaMKIINα. The dephosphorylation was carried out at 30°C in presence of reaction buffer containing 50 mM HEPES, 100 mM NaCl, 2 mM DTT, 0.01% Brij 35, pH 7.5, 1 mM MnCl_2_ and 0.001 mg/ml PP1 for 20 minutes. The dephosphorylation was stopped by adding SDS sample buffer. The concentration of CaMKII after staurosporine addition was 0.162 mg/ml, after GST-CaMKIINα addition was 0.082 mg/ml and before PP1 addition was 0.065 mg/ml. Staurosporine concentration after adding GST or GST-CaMKIINα to the reaction mix was 4.9 μM and before adding PP1 was 4 μM.

In the experiments to find the rate of dephosphorylation, autophosphorylation of CaMKII was carried out in a reaction mix containing 50 mM Tris, pH 8.0, 10 mM MgCl_2_, 10 mM DTT, 1 μM γ^32^P-ATP, 3 mM CaCl_2_, 9 μM calmodulin andCaMKII. For CaMKII expressed in HEK-293 cells 22 μM calmodulin was used. The concentrations of purified CaMKII used for autophosphorylation were 0.0081 mg/ml and 0.0087 mg/ml respectively for WT and E96A. The concentrations of HEK-293 cell lysates used were 2.21 mg/ml for WT, 1.46 mg/ml for H282A and 1.35 mg/ml for K21A. The reaction mix was preincubated at 30°C for 1 min and autophosphorylation was carried out for 5 minutes for WT and 20 minutes for E96A expressed in insect cells. For GFP-α-CaMKII expressed in HEK-293 cells, reaction was carried out for 5 minutes each for WT, H282A and K21A. The reaction was stopped with 10 μM staurosporine and non-radioactive ATP was added to a final concentration of 1 mM. The reaction mix was incubated with either 10 μM of GST-S1291A-GluN2A or GST-S1303A-GluN2B in presence of 22 μM calmodulin and 1 mM CaCl_2_ for 20 minutes at 4°C. For dephosphorylation, a buffer of pH 7.5 was added which contributed the following components at the indicated final concentrations: 50 mM HEPES, 100 mM NaCl, 2 mM DTT, 0.01% Brij 35 and 1 mM MnCl_2_. PP1 was added to a final concentration of 0.002 mg/ml and the reaction mix was incubated for the indicated times. For CaMKII expressed in HEK-293 cells, 0.0013–0.0033 mg/ml PP1 was used for dephosphorylation. The dephosphorylation was stopped by adding SDS sample buffer. The concentrations of the enzymes before PP1 addition were 0.0041 mg/ml and 0.0043 mg/ml for WT and E96A respectively and 1.11 mg/ml, 0.73 mg/ml, 0.68 mg/ml respectively for WT, H282A and K21A. Staurosporine concentration during incubation with GST-S1291A-GluN2A or GST-S1303A-GluN2B was 6.2 μM and before adding PP1 to the reaction mix was 5 μM. The reaction mix composition after addition of staurosporine became 49.5 mM Tris, 9.9 mM MgCl_2_, 9.9 mM DTT, 2.97 mM CaCl_2_; after addition of non-radioactive ATP became 48.5 mM Tris, 9.71 mM MgCl_2_, 9.71 mM DTT, 2.91 mM CaCl_2;_ after addition of calmodulin became 41.2 mM Tris, 8.24 mM MgCl_2_, 8.24 mM DTT, 2.47 mM CaCl_2;_ before addition of PP1 became 24.7 mM Tris, 4.95 mM MgCl_2_, 4.95 mM DTT, 1.48 mM CaCl_2;_ 50 mM HEPES, 100 mM NaCl, 2 mM DTT, 0.01% Brij 35, and 1 mM MnCl_2_. The samples were subjected to SDS-PAGE. The autoradiographic image of the gel was captured by phosphor imager. Phosphor image of the gel was quantitated using Quantity One software (Bio-Rad).

## 3. Results

### 3.1. GST-CaMKIINα inhibits dephosphorylation of CaMKII

**Fig 1:**
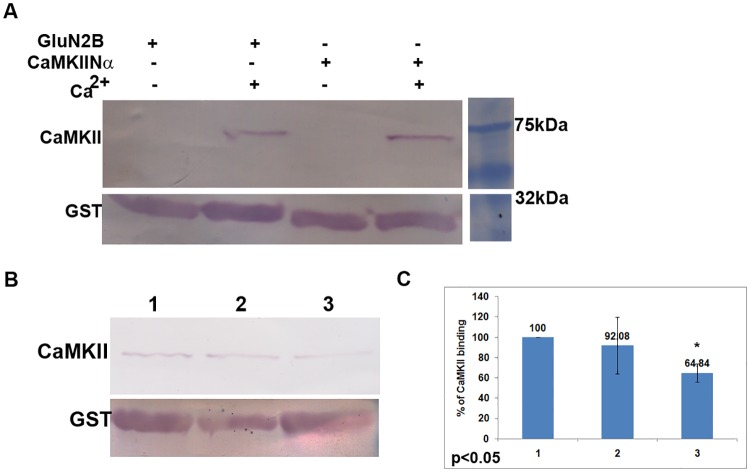
Calcium dependent binding of CaMKIINα to CaMKII. A: GST-pulldown assay was carried out to show binding of α-CaMKII to GST-CaMKIINα. Blot was cut into two pieces. Upper part of the blot was probed with anti-α-CaMKII antibody and the lower part was probed with anti-GST antibody. Marker is from the same blot. S1 Fig shows SDS-PAGE pattern of CaMKIINα. B: GluN2B reduced CaMKII binding to CaMKIINα. GST-pulldown assay was performed as described in methods. Lane 1: GST-pulldown of CaMKII with GST-CaMKIINα. Lane 2: CaMKIINα was allowed to bind to CaMKII first, followed by incubation with His-GluN2B. Lane 3: GST-CaMKIINα and His-GluN2B were allowed to bind to CaMKII simultaneously. C: The mean ± standard deviation of densitometric values of the band intensities obtained from three independent experiments as shown in B are plotted. The binding of CaMKII with GST-CaMKIINα taken as the control is considered as 100% binding. The binding of CaMKII in 2 and 3 are presented as the relative binding with respect to control in the bar graphs. The p value for the difference between 1 and 3 is less than 0.05 in two-tailed student’s t-test.

**Fig 2:**
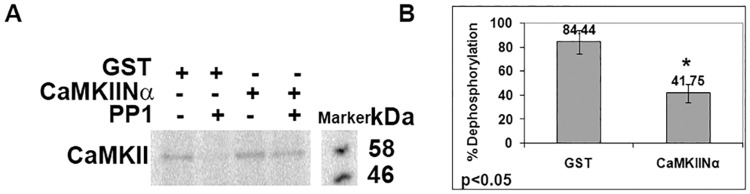
CaMKIINα inhibits the dephosphorylation of CaMKII. A: Autophosphorylated CaMKII was dephosphorylated in presence of either GST as control or GST-CaMKIINα. The same dephosphorylation condition was applied to all samples but, with and without treatment of PP1. B: Difference between the densitometric values of autoradiographic bands obtained with and without PP1 was used for calculating dephosphorylation. The values plotted are the mean ± standard deviation of data of four experiments. In each set, the intensity of the band without PP1 treatment was taken as 100% phosphorylation (p < 0.05 in two-tailed student’s t-test).

As reported earlier [34,42] we also find that CaMKII binds to GST-CaMKIINα inhibitor protein similar to its binding to GST-GluN2B (Fig 1A). We find that the binding of GST-CaMKIINα to CaMKII after 20 min incubation is tight and irreversible as His-GluN2B, even after one hour of incubation, could not replace the bound GST-CaMKIINα (Fig 1B). In a competition experiment, His-GluN2B competes with GST-CaMKIINα for the T-site of CaMKII (Fig 1B). Since CaMKIINα binds to the T-site of CaMKII similar to GluN2B, we checked whether it influences dephosphorylation of phospho-Thr^286^ of α-CaMKII. Phosphorylation at Thr^286^ and its dephosphorylation by PP1 were demonstrated under our experimental conditions using antibody specific to phospho-Thr^286^-α-CaMKII (S2 Fig). For dephosphorylation experiments, autophosphorylation of CaMKII, carried out using γ^32^P-ATP, was stopped with staurosporine to prevent any further kinase reaction. It was found that the dephosphorylation of CaMKII by PP1 was reduced in presence of GST-CaMKIINα inhibitor protein (Fig 2).

### 3.2. Glu^96^ and His^282^ are key residues involved in mediating the GluN2B induced effects on CaMKII dephosphorylation

**Fig 3:**
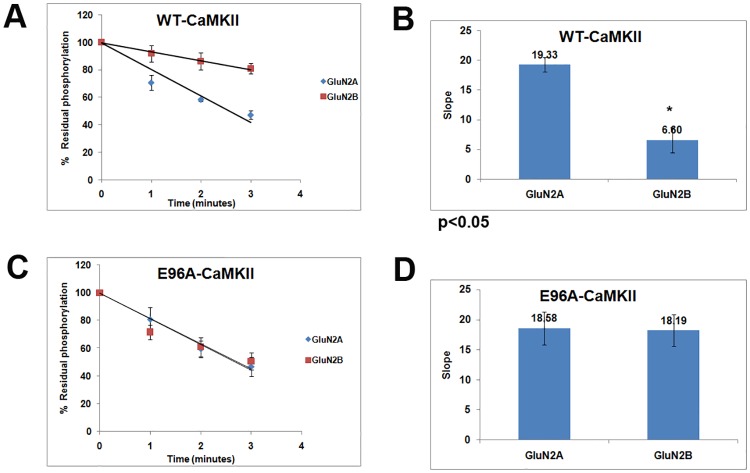
Comparison of the effect of GluN2B on the rates of dephosphorylation of WT and E96A. A and C: The extent of dephosphorylation of WT and E96A mutant of α-CaMKII were determined at 1, 2 and 3 minutes in presence of GST-S1303A-GluN2B or GST-S1291A-GluN2A. Phosphorylation intensity without PP1 at 0 time was taken as 100%. The values plotted were from the densitometric data of autoradiographic images as shown in S5 Fig, from three experiments. B and D: The slope values of the plots of the time course of dephosphorylation in presence of GluN2B and GluN2A sequences are presented as the bar graphs (For B, p<0.05 in a two—tailed student’s t-test).

The dephosphorylation of CaMKII had been shown to be inhibited by GST-S1303A-GluN2B [9]. Here we show that the rate of dephosphorylation of WT-CaMKII in presence of GST-S1303A-GluN2B was slower compared to that in presence of GST-S1291A-GluN2A (Fig 3 and S5 Fig). The rate of dephosphorylation is calculated from the slope of the plot of phosphorylation level against the time. About three fold decrease in the rate of dephosphorylation of WT-CaMKII was observed in presence of GST-S1303A-GluN2B.

The position of Glu^96^ of α-CaMKII or its analogous residues in other isoforms of CaMKII in the active and autoinhibited states suggests that this residue is involved in ATP binding. The movement of the D-helix in the active structure swings Glu^96^ towards ATP binding site which is positioned away from the ATP binding site in the autoinhibited state [43,44]. In the CaMKIIδ/Ca^2+^/CaM complex Glu^97^ (Glu^96^ in α-CaMKII) is suitably positioned to coordinate with ATP [44]. We have previously shown that in the E96A-α-CaMKII mutant, GluN2B binding-induced modulation of catalysis that is seen in WT-α-CaMKII, is impaired [27] showing that Glu^96^ has the additional role of mediating the GluN2B induced structural changes. E96A-α-CaMKII binds to GluN2B although the extent of binding was modestly different compared to WT (S6 Fig). The binding to GluN2B by E96A was comparable to WT after autophosphorylation at Thr^286^ (S7 Fig). When the dephosphorylation of E96A-α-CaMKII was analysed, it was found that inhibition of dephosphorylation by GluN2B seen in WT was almost completely absent in the E96A mutant indicating that the GluN2B binding induced alterations in the structure of CaMKII did not occur in the mutant (Fig 3 and S5 Fig). This suggested that the same structural changes mediated by Glu^96^ of α-CaMKII upon binding of GluN2B may be responsible for catalytic modulation [27] as well as reduction of Thr^286^ dephosphorylation rate.

**Fig 4:**
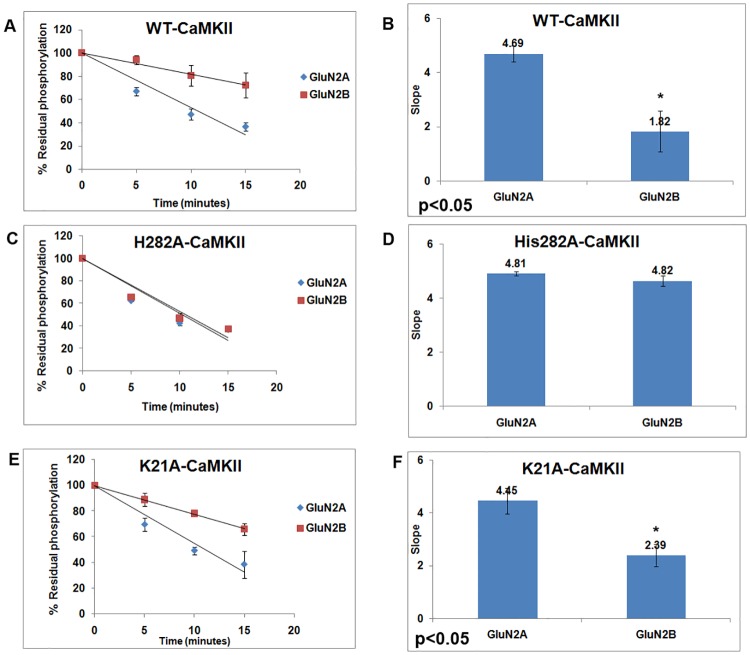
Comparison of the effect of GluN2B on the rates of dephosphorylation of WT, H282A and K21A forms of α-CaMKII. WT and mutants, H282A and K21A, of α-CaMKII expressed with N-terminal GFP fusions in HEK-293 cells were used for the experiments. A, C and E: The time course of dephosphorylation of WT, H282A and K21A in presence of GST-S1303A-GluN2B or GST-S1291A-GluN2A at 5, 10 and 15 minutes. Phosphorylation intensity without PP1 at 0 time was taken as 100%. The values plotted were from the densitometric data of autoradiographic images as shown in S5 Fig, from three experiments. B, D and F: The slope values of the plots of the time course of dephosphorylation in presence of GluN2B and GluN2A sequences are presented as the bar graphs. (For B and F, p<0.05 in a two-tailed student’s t-test)

We checked the dephosphorylation rate of another mutant, H282A-α-CaMKII. His^282^ is a residue involved in anchoring the D-helix of α-CaMKII [43]. H282A mutation is reported to bring about interesting biochemical consequences in CaMKII [45,46]. Since His^282^ is also involved in maintaining the structure of D-helix similar to Glu^96^, we anticipated functional anomalies in the H282A-α-CaMKII mutant similar to the E96A mutant. We found that H282A-α-CaMKII exhibited Ca^2+^-independence for its autophosphorylation activity (S2 Fig) as well as for binding to GluN2B (data not shown). However its GluN2B binding showed specificity when compared to GluN2A (S3 Fig). Moreover, the mutant was comparable to WT for its binding to GluN2B whether it was autophosphorylated or not (S6 and S7 Figs). When Thr^286^dephosphorylation was studied, it was found that H282A-α-CaMKII also behaved like E96A mutant and showed similar rate of dephosphorylation with or without GluN2B (Fig 4 and S5 Fig). Lys^21^ is a residue in the ATP binding region. GluN2B binds to K21A-α-CaMKII mutant in its autophosphorylated and non-autophosphorylated states as seen in the pulldown assay (S4, S6 and S7 Figs). The K21A-α-CaMKII mutant showed decrease in the rate of dephosphorylation in presence of GluN2B compared to that in presence of GluN2A (Fig 4 and S5 Fig) showing that Lys^21^ does not appear to be involved in the GluN2B-mediated effects and that the effects seen with Glu^96^ and His^282^ are specific.

## 4. Discussion

CaMKII functions as a molecular switch supporting memory in PSD and a fine balance between the kinase and phosphatase is required for optimal functioning of the switch [13–15]. As reported earlier, we see that CaMKIINα binds to the T-site of CaMKII [34]. We also show that the binding of CaMKIINα inhibitor protein is irreversible and persistent even after calcium removal. GluN2B sequence inhibits the binding of CaMKIINα to CaMKII indicating that both could be interacting at the T-site. CaMKIINα may be involved in a feedback mechanism in CaMKII signaling in memory formation [47].

Lisman *et al* hypothesized that CaMKII-PP1 system may be acting as a bistable molecular switch forming the basis for molecular memory [13]. Both LTP and LTD, the two forms of synaptic plasticity are mediated by phospho-Thr^286^-CaMKII autonomous activity [48]. CaMKII-GluN2B interaction is required for LTP induction as well as maintenance of synaptic strength [49]. GluN2B interaction induced catalytic modulation of CaMKII stabilizes its activity against fluctuations in ATP concentrations [9,27]. Restrained dephosphorylation of phospho-Thr^286^-CaMKII when it is bound to GluN2B *in vitro* or in PSD [9,50] is considered as a fundamental characteristic of the CaMKII-GluN2B complex that allows it to support the maintenance of LTP [30]. In the crystal structure of the truncated CaMKII, regulatory region of one subunit binds to the active site of the neighbouring subunit. The N-terminal portion of the regulatory region (amino acids 276–291) of one subunit interacts with the C-lobe of the neighbouring kinase subunit. In the active state conformation, Arg^283^-Leu^291^ of the regulatory region of the subunit acting as substrate binds to the active site of the enzyme subunit [51]. Arg^283^ interacts electrostatically with an acidic region in the enzyme subunit positioning Thr^286^ into the active site [51]. It has been proposed that Thr^286^ of α-CaMKII in the phosphorylated state might also occupy the active site of the neighbouring subunit. When phospho-Thr^286^ is bound to the active site it could be inaccessible to phosphatase. GluN2B interaction might facilitate this binding thereby reducing dephosphorylation by PP1 [30]. His^282^ being close to Arg^283^, is likely to be involved in electrostatic interactions with docking sites in the acidic region of the enzyme subunit. Mutation of His^282^ to Ala might destabilize these interactions thereby exposing phospho-Thr^286^ to the action of phosphatase. Glu^96^ mutation to Ala might also perturb this interaction and expose phospho-Thr^286^ to phosphatase consistent with our data presented here. Indeed PP1 has been shown to be a suppressor of learning and memory [52]. Hence in addition to GluN2B, other endogenous T-site binding proteins that may also regulate the accessibility of the phosphatase to CaMKII could play a supportive role for the proper functioning of the CaMKII-PP1 switch. Our finding reported in the current manuscript that CaMKIINα inhibits dephosphorylation of phospho-Thr^286^-CaMKII suggests that CaMKIINα may thus support the CaMKII-PP1 molecular switch.

The dual consequences of the binding of GluN2B to the T-site of CaMKII described above, i.e. catalytic modulation and inhibition of dephosphorylation, could be the result of the same structural mechanism. We have previously shown that in the E96A mutant of α-CaMKII, the catalytic modulation is impaired eventhough the mutant binds GluN2B [27] indicating that Glu^96^ could be playing a role in the associated structural changes. The present study shows that this mutant is defective in exhibiting the GluN2B-induced reduction in dephosphorylation also (Fig 3) suggesting that the same structural mechanisms could be responsible for both the effects. In the inactive state of CaMKII the regulatory segment interacts to the helix αD which positions Glu^96^ away from the ATP binding site [43]. In the active state as the regulatory segment disengages, helix αD moves towards ATP so that Glu^96^ (Glu^97^ in δ-CaMKII) interacts with the hydroxyl groups of ribose rings of ATP [44]. We hypothesise that Glu^96^ of CaMKII might be participating in essential interactions that stabilize the GluN2B-induced altered conformation. His^282^ is also involved in anchoring D helix of α-CaMKII [43]. As expected, H282A-α-CaMKII showed similar results like E96A mutant in the regulation of dephosphorylation. It appears that modulation of catalysis of CaMKII as well as inhibition of dephosphorylation of phospho-Thr^286^-CaMKII are both consequences of the same set of structural changes in CaMKII that are induced by binding of ligands at the T-site.

This study reveals the commonality and cooperation of the functions of two key residues of α-CaMKII, Glu^96^ and His^282^ and also provides insights for further investigations on the regulation of CaMKII functions by GluN2B. The data on CaMKIINα also suggests additional physiological roles for this inhibitory protein of CaMKII.

## Supporting Information

S1 Fig SDS-PAGE of the purified GST-S1303A-GluN2B, GST-S1291A-GluN2A, and GST-CaMKIINα. (TIF)

S2 Fig Autophosphorylation of Thr^286^ and its dephosphorylation by PP1. Autophosphorylation and dephosphorylation of Thr^286^ were monitored by Western blotting using phospho-Thr^286^-specific antibodies. **A:** Thr^286^ autophosphorylation of CaMKII enzymes were carried out as in the methods section 2.5. The upper panel shows the Western blot of the non-autophosphorylated and autophosphorylated purified WT-α-CaMKII (5.4 μg) and E96A-α-CaMKII (14 μg) that was expressed in insect cells probed with anti-phospho-Thr^286^-α-CaMKII antibody. We note that the band intensity in the CaMKII blot for the mutant is lesser than expected. For non-autophosphorylated samples ATP was added after SDS sample buffer addition. ATP-dependent autophosphorylation was observed. The lower panel shows the same blot probed with anti-α-CaMKII antibody. There is only slight band shift between autophosphorylated and non-autophosphorylated samples indicating that no sites other than Thr^286^ site is autophosphorylated under the reaction conditions. E96A-α-CaMKII band was seen at slightly higher position than WT-α-CaMKII. B: The upper panel shows the Western blot of the non-autophosphorylated and autophosphorylated HEK-293 cell lysates expressing GFP-WT-α-CaMKII (28 μg), GFP-K21A-α-CaMKII (35 μg) and GFP-H282A-α-CaMKII (28 μg) probed with anti-phospho-Thr^286^-α-CaMKII antibody. Since HEK-293 cells have endogenous ATP as reported before [53] negative control for autophosphorylation was carried out without Ca^2+^. The lysates without any incubation were also directly loaded. H282A-α-CaMKII shows Ca^2+^ independent activity. The lower panel shows the same blot probed with anti-α-CaMKII antibody. C: Protein phosphatase 1 dephosphorylates phospho-Thr^286^-α-CaMKII *in vitro*. Western blot shows CaMKII-Thr^286^ autophosphorylation and its dephosphorylation by PP1. Data represents two experiments.
(TIF)

S3 Fig GFP-H282A-α-CaMKII binds to GluN2B. GST-pulldown was carried out for WT and H282A mutant of α-CaMKII expressed as GFP-fusion in HEK-293 cells. GST-GluN2A fusion protein was used as negative control. Lane 1: H282A-α-CaMKII with GluN2A in presence of calcium. Lane 2: WT-α-CaMKII with GluN2A in presence of calcium. Lane 3: H282A-α-CaMKII with GluN2B in presence of calcium. Lane 4: WT-α-CaMKII with GluN2B in presence of calcium. Lane 5: Molecular size markers. The upper panel shows the blot probed with anti-α-CaMKII antibody and lower panel shows the blot probed with anti-GST antibody. The lanes from the same blot are presented.
(TIF)

S4 Fig GFP-K21A-α-CaMKII binds to GluN2B. Lane 1: GST-pulldown of K21A-α-CaMKII expressed as GFP-fusion in HEK-293 cells with GluN2B in presence of calcium. Lane 2: GST-pull-down of K21A-α-CaMKII with GluN2A in presence of calcium. Lane 3: Molecular size marker.
(TIF)

S5 Fig Comparison of the rates of dephosphorylation of WT and mutants of α-CaMKII. Autoradiographic images showing the band corresponding to α-CaMKII phosphorylated at Thr^286^ with ^32^P and the dephosphorylation by PP1 of the different enzymes at the indicated times in the presence of either GluN2A serving as the control or in the presence of GluN2B. Autophosphorylated enzymes before PP1 additions were taken as the 0 time point samples. A: CaMKII purified after expression in insect cells was used for the experiments. The upper panel shows the autoradiographic image of the dephosphorylation pattern of WT-α-CaMKII. The lower panel shows the dephosphorylation pattern of the E96A-α-CaMKII. B: CaMKII expressed as GFP-fusion in HEK-293 cells were used for the experiments. The upper panel shows the autoradiographic image of the dephosphorylation pattern of GFP-WT-α-CaMKII. The middle panel shows dephosphorylation pattern of GFP-H282A-α-CaMKII and the lower panel shows the dephosphorylation pattern of GFP-K21A-α-CaMKII.
(TIF)

S6 Fig Mutants of CaMKII bind to GluN2B. A and B: WT and E96A forms of α-CaMKII expressed in insect cells bind to GluN2B. The input, bound CaMKII in the pellet and the supernatant of the GST pulldown assay were subjected to Western blot analysis. Blots presented in A and B were developed using alkaline phosphatase conjugated secondary antibody. A: The input of WT and E96A used in the GST-pulldown assay with GST-GluN2B and the complete supernatant of unbound enzyme of the GST-pulldown assay. B: The upper panel shows the bound WT and E96A in the pellet as detected by α-CaMKII antibody and the lower panel indicates the GST-GluN2B as detected by GST antibody. C: The bar graphs indicate the mean ± standard deviation (n = 3) of bound WT and E96A normalized to band intensities of GST-GluN2B and to input, as quantified by densitometry in a Biorad Versadoc gel documentation system and Biorad Quantity One software. p = 0.0006 in a two—tailed student’s t-test. D, E and F: WT, K21A and H282A-α-CaMKII expressed as GFP-fusions in HEK-293 cells bind to GluN2B. D: The input of WT, K21A and H282A used and half of the supernatant of unbound enzymes of the GST-pulldown assay. E. The upper panel shows the bound WT, K21A and H282A in the pellet as detected by α-CaMKII antibody and the lower panel indicates the GST-GluN2B as detected by GST antibody. F: The bar graphs indicate the mean ± standard deviation (n = 3) of bound WT, K21A and H282A quantified by densitometry. The p values were calculated in a two—tailed student’s t-test for the difference between WT and K21A (p = 0.034) and WT and H282A (p = 0.0034).
(TIF)

S7 Fig Autophosphorylated WT and mutants of CaMKII bind to GluN2B. A and B: Autophosphorylated WT and E96A forms of α-CaMKII expressed in insect cells bind to GST-GluN2B. Western blot analysis of purified autophosphorylated WT and E96A expressed in insect cells used as the input, the supernatant of unbound enzyme and the bound enzyme in the GST pulldown assay are shown. A: WT and E96A input and complete supernatant of the GST-pulldown assay. B: The upper panel shows the bound autophosphorylated WT and E96A in the GST pulldown assay with GST-GluN2B as detected by α-CaMKII antibody. The lower panel indicates GST-GluN2B probed by GST antibody. C: The bar graphs shows the mean ± standard deviation (n = 3) of the bound autophosphorylated WT and E96A quantified by densitometry of the bands and normalized as indicated. p = 0.0006 in a two-tailed student’s t-test. D, E and F: Autophosphorylated WT, K21A and H282A forms of α-CaMKII expressed as GFP-fusion in HEK-293 cells bind to GluN2B. D: The input of WT, K21A and H282A and half of the supernatant of unbound enzymes of the GST-pulldown assay with GluN2B. E. The upper panel shows the bound WT, K21A and H282A in the pellet as detected by α-CaMKII antibody and the lower panel indicates the GST-GluN2B as detected by GST antibody. F: The bar graphs indicate the mean ± standard deviation (n = 3) of the amount of bound WT, K21A and H282A obtained by densitometric quantification of the bands. The p values were calculated in a two—tailed student’s t-test for the difference between WT and K21A (p = 0.40) and WT and H282A (p = 0.43).
(TIF)
