# Supplementary figures and images for: Protection of α-CaMKII from Dephosphorylation by GluN2B Subunit of NMDA Receptor Is Abolished by Mutation of Glu96 or His282 of α-CaMKII
Source: PLoS One. 2016 Sep 9;11(9):e0162011. doi: 10.1371/journal.pone.0162011 (PMC5017783; doi:10.1371/journal.pone.0162011)

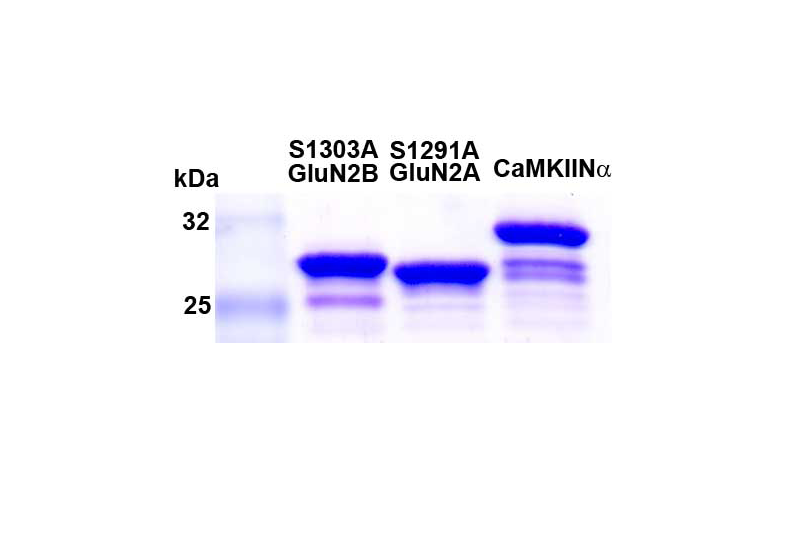

Supplement: S1 Fig — (TIF) [file pone.0162011.s001.tif]

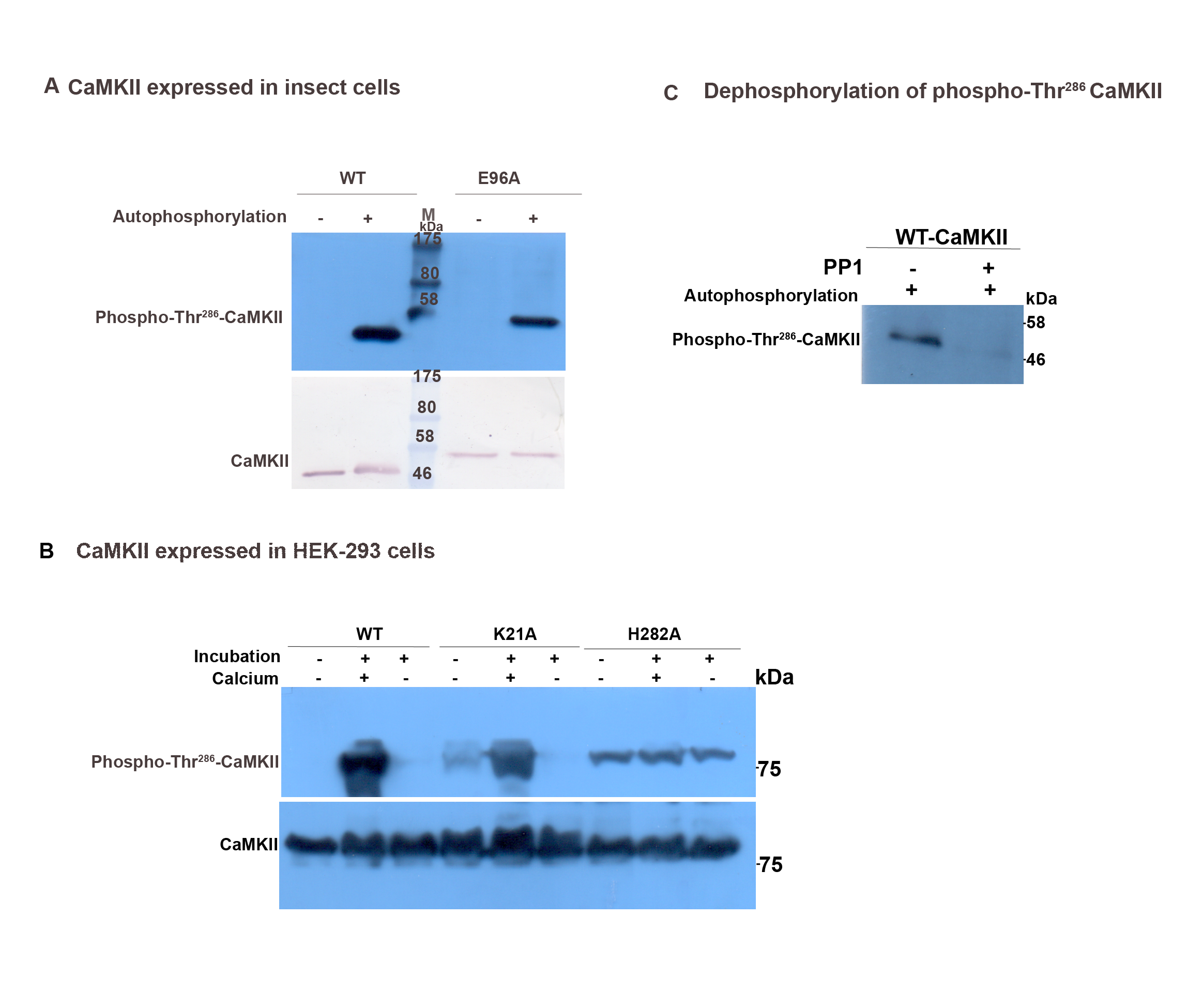

Supplement: S2 Fig — Autophosphorylation and dephosphorylation of Thr286 were monitored by Western blotting using phospho-Thr286-specific antibodies. A: Thr286 autophosphorylation of CaMKII enzymes were carried out as in the methods section 2.5. The upper panel shows the Western blot of the non-autophosphorylated and autophosphorylated purified WT-α-CaMKII (5.4 μg) and E96A-α-CaMKII (14 μg) that was expressed in insect cells probed with anti-phospho-Thr286-α-CaMKII antibody. We note that the band intensity in the CaMKII blot for the mutant is lesser than expected. For non-autophosphorylated samples ATP was added after SDS sample buffer addition. ATP-dependent autophosphorylation was observed. The lower panel shows the same blot probed with anti-α-CaMKII antibody. There is only slight band shift between autophosphorylated and non-autophosphorylated samples indicating that no sites other than Thr286 site is autophosphorylated under the reaction conditions. E96A-α-CaMKII band was seen at slightly higher position than WT-α-CaMKII. B: The upper panel shows the Western blot of the non-autophosphorylated and autophosphorylated HEK-293 cell lysates expressing GFP-WT-α-CaMKII (28 μg), GFP-K21A-α-CaMKII (35 μg) and GFP-H282A-α-CaMKII (28 μg) probed with anti-phospho-Thr286-α-CaMKII antibody. Since HEK-293 cells have endogenous ATP as reported before [53] negative control for autophosphorylation was carried out without Ca2+. The lysates without any incubation were also directly loaded. H282A-α-CaMKII shows Ca2+ independent activity. The lower panel shows the same blot probed with anti-α-CaMKII antibody. C: Protein phosphatase 1 dephosphorylates phospho-Thr286-α-CaMKII in vitro. Western blot shows CaMKII-Thr286 autophosphorylation and its dephosphorylation by PP1. Data represents two experiments. (TIF) [file pone.0162011.s002.tif]

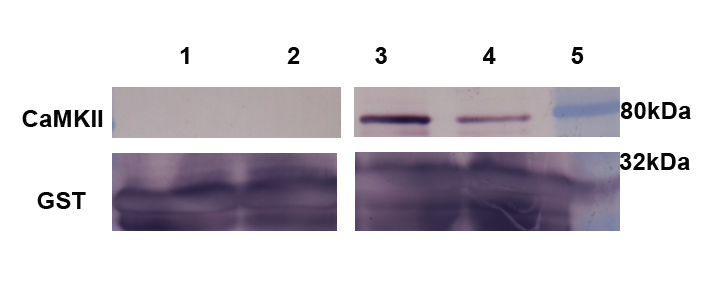

Supplement: S3 Fig — GST-pulldown was carried out for WT and H282A mutant of α-CaMKII expressed as GFP-fusion in HEK-293 cells. GST-GluN2A fusion protein was used as negative control. Lane 1: H282A-α-CaMKII with GluN2A in presence of calcium. Lane 2: WT-α-CaMKII with GluN2A in presence of calcium. Lane 3: H282A-α-CaMKII with GluN2B in presence of calcium. Lane 4: WT-α-CaMKII with GluN2B in presence of calcium. Lane 5: Molecular size markers. The upper panel shows the blot probed with anti-α-CaMKII antibody and lower panel shows the blot probed with anti-GST antibody. The lanes from the same blot are presented. (TIF) [file pone.0162011.s003.tif]

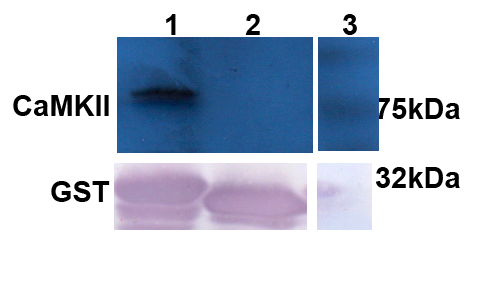

Supplement: S4 Fig — Lane 1: GST-pulldown of K21A-α-CaMKII expressed as GFP-fusion in HEK-293 cells with GluN2B in presence of calcium. Lane 2: GST-pull-down of K21A-α-CaMKII with GluN2A in presence of calcium. Lane 3: Molecular size marker. (TIF) [file pone.0162011.s004.tif]

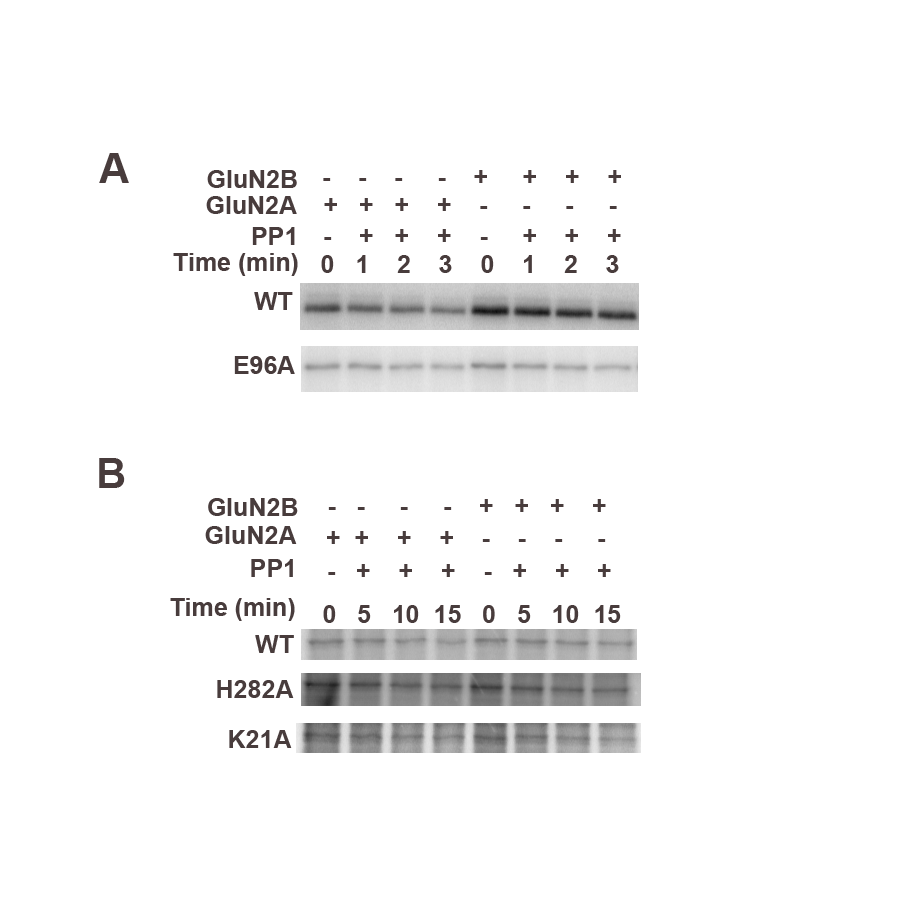

Supplement: S5 Fig — Autoradiographic images showing the band corresponding to α-CaMKII phosphorylated at Thr286 with 32P and the dephosphorylation by PP1 of the different enzymes at the indicated times in the presence of either GluN2A serving as the control or in the presence of GluN2B. Autophosphorylated enzymes before PP1 additions were taken as the 0 time point samples. A: CaMKII purified after expression in insect cells was used for the experiments. The upper panel shows the autoradiographic image of the dephosphorylation pattern of WT-α-CaMKII. The lower panel shows the dephosphorylation pattern of the E96A-α-CaMKII. B: CaMKII expressed as GFP-fusion in HEK-293 cells were used for the experiments. The upper panel shows the autoradiographic image of the dephosphorylation pattern of GFP-WT-α-CaMKII. The middle panel shows dephosphorylation pattern of GFP-H282A-α-CaMKII and the lower panel shows the dephosphorylation pattern of GFP-K21A-α-CaMKII. (TIF) [file pone.0162011.s005.tif]

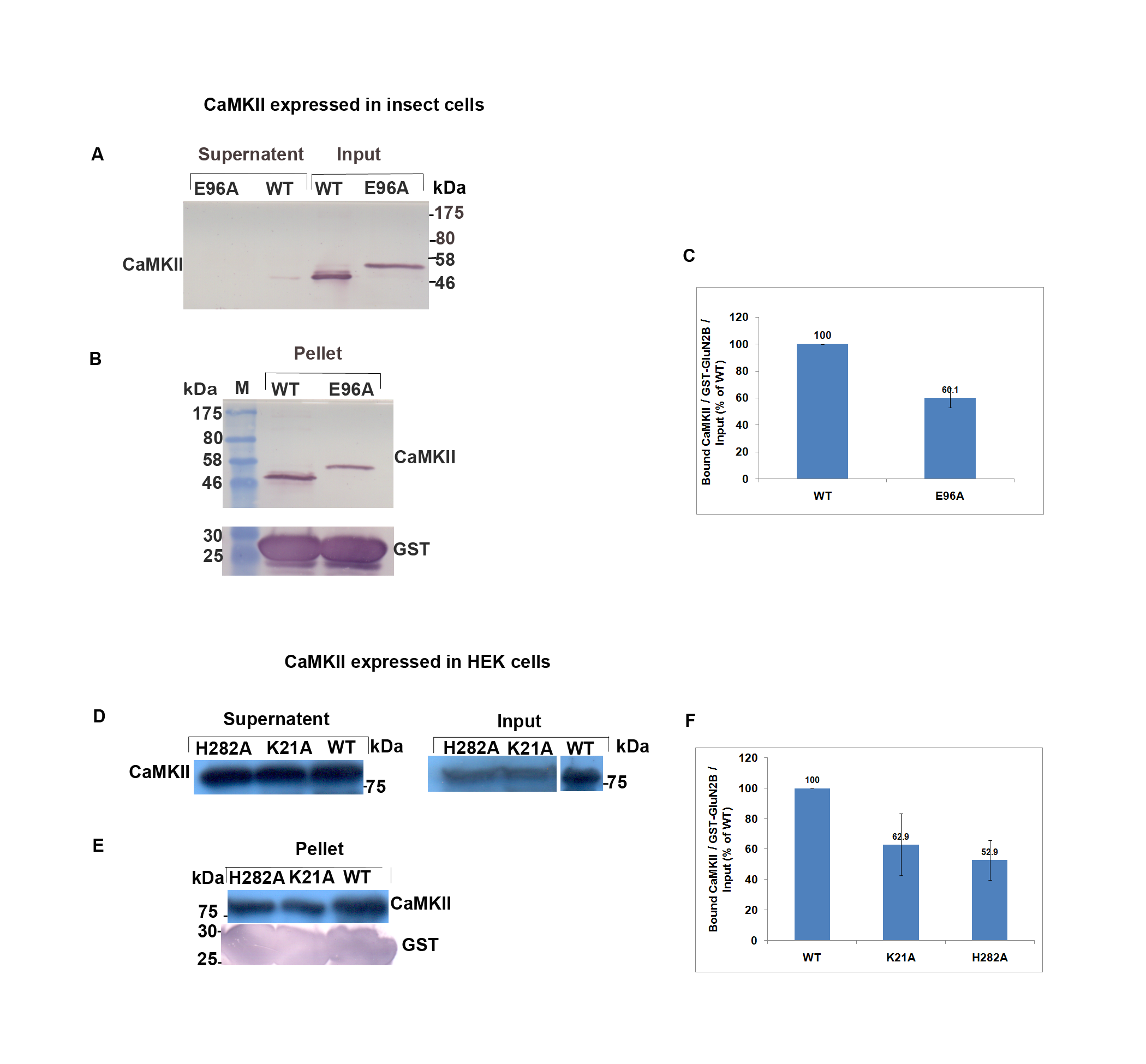

Supplement: S6 Fig — A and B: WT and E96A forms of α-CaMKII expressed in insect cells bind to GluN2B. The input, bound CaMKII in the pellet and the supernatant of the GST pulldown assay were subjected to Western blot analysis. Blots presented in A and B were developed using alkaline phosphatase conjugated secondary antibody. A: The input of WT and E96A used in the GST-pulldown assay with GST-GluN2B and the complete supernatant of unbound enzyme of the GST-pulldown assay. B: The upper panel shows the bound WT and E96A in the pellet as detected by α-CaMKII antibody and the lower panel indicates the GST-GluN2B as detected by GST antibody. C: The bar graphs indicate the mean ± standard deviation (n = 3) of bound WT and E96A normalized to band intensities of GST-GluN2B and to input, as quantified by densitometry in a Biorad Versadoc gel documentation system and Biorad Quantity One software. p = 0.0006 in a two—tailed student’s t-test. D, E and F: WT, K21A and H282A-α-CaMKII expressed as GFP-fusions in HEK-293 cells bind to GluN2B. D: The input of WT, K21A and H282A used and half of the supernatant of unbound enzymes of the GST-pulldown assay. E. The upper panel shows the bound WT, K21A and H282A in the pellet as detected by α-CaMKII antibody and the lower panel indicates the GST-GluN2B as detected by GST antibody. F: The bar graphs indicate the mean ± standard deviation (n = 3) of bound WT, K21A and H282A quantified by densitometry. The p values were calculated in a two—tailed student’s t-test for the difference between WT and K21A (p = 0.034) and WT and H282A (p = 0.0034). (TIF) [file pone.0162011.s006.tif]

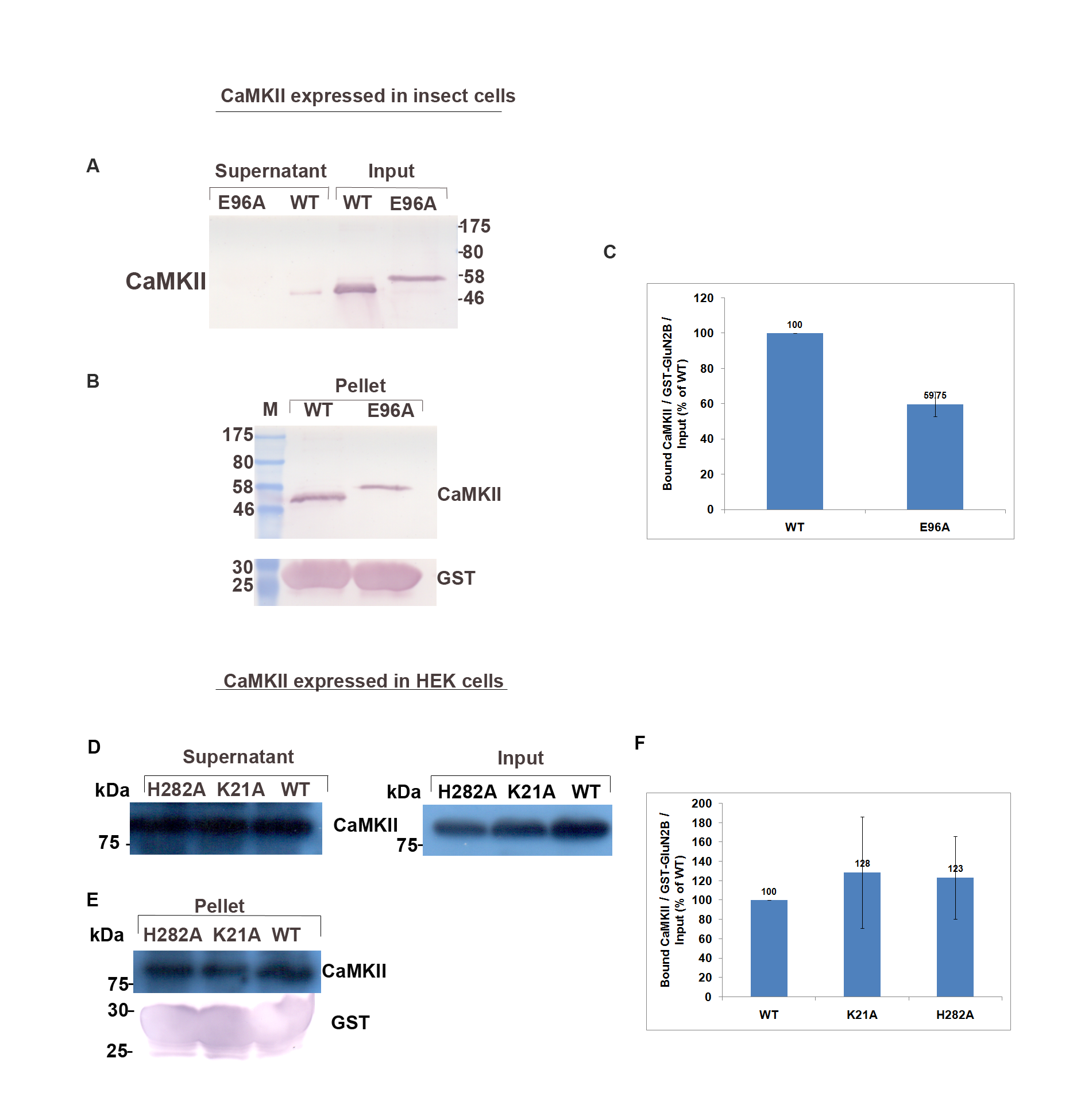

Supplement: S7 Fig — A and B: Autophosphorylated WT and E96A forms of α-CaMKII expressed in insect cells bind to GST-GluN2B. Western blot analysis of purified autophosphorylated WT and E96A expressed in insect cells used as the input, the supernatant of unbound enzyme and the bound enzyme in the GST pulldown assay are shown. A: WT and E96A input and complete supernatant of the GST-pulldown assay. B: The upper panel shows the bound autophosphorylated WT and E96A in the GST pulldown assay with GST-GluN2B as detected by α-CaMKII antibody. The lower panel indicates GST-GluN2B probed by GST antibody. C: The bar graphs shows the mean ± standard deviation (n = 3) of the bound autophosphorylated WT and E96A quantified by densitometry of the bands and normalized as indicated. p = 0.0006 in a two-tailed student’s t-test. D, E and F: Autophosphorylated WT, K21A and H282A forms of α-CaMKII expressed as GFP-fusion in HEK-293 cells bind to GluN2B. D: The input of WT, K21A and H282A and half of the supernatant of unbound enzymes of the GST-pulldown assay with GluN2B. E. The upper panel shows the bound WT, K21A and H282A in the pellet as detected by α-CaMKII antibody and the lower panel indicates the GST-GluN2B as detected by GST antibody. F: The bar graphs indicate the mean ± standard deviation (n = 3) of the amount of bound WT, K21A and H282A obtained by densitometric quantification of the bands. The p values were calculated in a two—tailed student’s t-test for the difference between WT and K21A (p = 0.40) and WT and H282A (p = 0.43). (TIF) [file pone.0162011.s007.tif]
